# Synergistic Meropenem/Vaborbactam Plus Fosfomycin Treatment of KPC Producing *K. pneumoniae* Septic Thrombosis Unresponsive to Ceftazidime/Avibactam: From the Bench to the Bedside

**DOI:** 10.3390/antibiotics10070781

**Published:** 2021-06-27

**Authors:** Alessandra Oliva, Ambrogio Curtolo, Lorenzo Volpicelli, Francesco Cogliati Dezza, Massimiliano De Angelis, Sara Cairoli, Donatella Dell’Utri, Bianca Maria Goffredo, Giammarco Raponi, Mario Venditti

**Affiliations:** 1 Department of Public Health and Infectious Diseases, Sapienza University of Rome, 00185 Rome, Italy; alessandra.oliva@uniroma1.it (A.O.); ambrogio.curt@uniroma1.it (A.C.); lorenzo.volpicelli@uniroma1.it (L.V.); francesco.cogliatidezza@uniroma1.it (F.C.D.); massimiliano.deangelis@uniroma1.it (M.D.A.); giammarco.raponi@uniroma1.it (G.R.); 2 Biochemistry Laboratory, Department of Specialist Pediatrics, Bambino Gesù Children’s Hospital, 00165 Rome, Italy; sara.cairoli@opbg.net (S.C.); biancamaria.goffredo@opbg.net (B.M.G.); 3 Department of Anesthesia and Critical Care Medicine, Policlinico Umberto I, 00161 Rome, Italy; donatella.dellutri@uniroma1.it

**Keywords:** septic thrombosis, meropenem/vaborbactam, fosfomycin, synergism, pharmacokinetic analyses

## Abstract

Gram-negative bacilli septic thrombosis (GNB-ST) represents a subtle and often misleading condition, potentially fatal if not recognized early and requiring prolonged antimicrobial therapy and anticoagulation. Herein, reported for the first time, is a very challenging case of *Klebsiella* producing carbapenemase (KPC)-producing *K. pneumoniae* (KPC-Kp) ST unresponsive to ceftazidime/avibactam (CZA) relapsed first with meropenem/vaborbactam (MVB) monotherapy and subsequently cured with MVB plus fosfomycin (FOS) combination. The present case highlights the possibility of CZA underexposure on the infected thrombus and the risk of in vivo emergence of CZA resistance in the setting of persistent bacteremia and sub-optimal anticoagulation. Pharmacokinetic analyses showed that both MVB and FOS were in the therapeutic range. In vitro studies demonstrated a high level of MVB + FOS synergism that possibly allowed definitive resolution of the endovascular infection.

## 1. Introduction

Septic thrombosis (ST) is a serious and potentially fatal condition defined by the presence of an endovascular thrombus in the setting of associated bacterial or fungal infection [1,2,3,4]. The cornerstones of ST treatment include prolonged and targeted antimicrobial therapy and anticoagulation [1,4,5]. Furthermore, in selected cases surgical debridement may also be required [6,7,8]. Of importance, multi-drug resistant organisms (MDRO) may cause ST; in particular, etiologies may include not only methicillin-resistant *Staphylococcus aureus* (MRSA) but also extended-spectrum beta-lactamase (ESBL)-producing and carbapenem-resistant *Enterobacterales* (CRE) [4,9,10,11].

Since 2017, the novel antimicrobial coformulation meropenem/vaborbactam (MVB), the latter being a cyclic boronic acid-based beta-lactamase competitive inhibitor specifically designed to inhibit KPC enzyme, addressed the need for agents with activity against CRE [12,13].

Herein, we report the case of successful treatment, first with MVB alone and subsequently with MVB plus fosfomycin (FOS), in a critically ill woman affected by KPC-Kp ST unresponsive to treatment with CZA. This case offers the opportunity to discuss the clinical complexity of ST management and the reasons for CZA substitution with MVB. Furthermore, since MVB was also used in combination with FOS, we had the opportunity to study in vitro synergism between these two antimicrobials. Pharmacokinetic analyses were also performed.

## 2. Case Description

**Figure 1:**
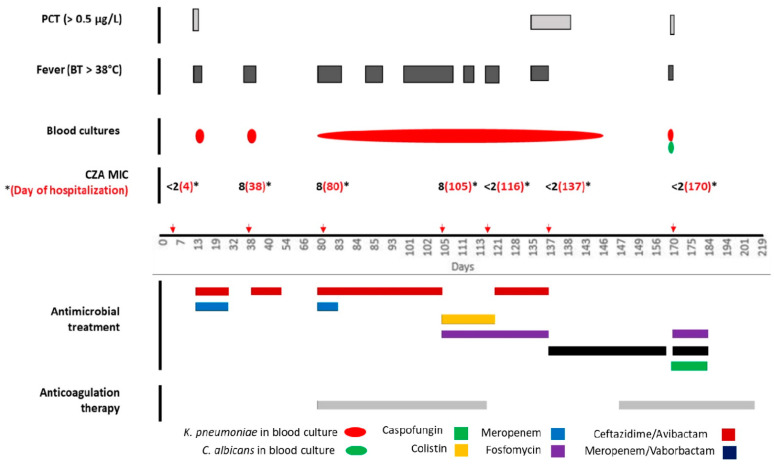
Timeline of the clinical, microbiological, and therapeutic course of the case. PCT: procalcitonin; BT: body temperature; CZA: ceftazidime/avibactam. Red arrows represent the exact day of KPC-Kp isolation from biological samples. The *x*-axis is not uniformly proportioned on purpose, since the extreme complexity of the case forced us to skip the correct proportion of days.

Details of the clinical course are shown in Figure 1 and data on sequential KPC-Kp isolates antibiotics susceptibilities are shown in Table 1. A 45-year-old woman was admitted to the Intensive Care Unit on 30 May 2020 (day 0) because of subarachnoid hemorrhage caused by aneurysm rupture of the middle cerebral artery treated with clipping surgery. On admission, she was neither conscious nor hemodynamically stable, and required vasopressors and mechanical ventilation. Her medical history showed hypothyroidism and obesity (Body Mass Index, 36). Four days after the admission a rectal swab for CRE resulted positive for a KPC-Kp. On day 13, she was pyretic with a body temperature (BT) of 39 °C and the blood cultures (BCs) documented a bloodstream infection (BSI) due to KPC-Kp with the same antimicrobial susceptibility pattern of that isolated from rectal swab and molecular analysis showed that the isolate was producing the KPC enzyme (Table 1).

A combination treatment with CZA 2.5 gr every 8 h (3-h infusion) and MEM 2 gr every 8 h (3-h infusion) was started. Follow-up BCs (FUBCs) yielded no growth and therefore on day 31 the antimicrobial therapy was interrupted. A week later, a KPC-Kp BSI relapse was documented and a new CZA monotherapy treatment course was started and continued for 14 days, with, again, prompt disappearance of fever and clearance of bacteremia. Rectal swab remained positive for KPC-Kp. On day 80, for a new KPC-Kp BSI relapse with persistence of bacteremia even after central venous catheter (CVC) removal, ST was suspected. A Doppler ultrasonography revealed a thrombosis lesion occluding the left subclavian vein and a parietal thrombosis in the right one, which were both confirmed at a subsequent CT angiography. Thus, CZA and anticoagulation therapy with low molecular weight heparin (LMWH) 8000 international units (UI) every 12 h were commenced.

On day 105, colistin (9 MU as loading dose followed by 4.5 MU every 12 h) plus FOS (8 gr every 8 h) replaced CZA because of persistently positive FUBCs for a KPC-Kp strain with reduced susceptibility to CZA (MIC = 8 μg/mL) (Figure 1). On day 115, the patient developed an acute expanding thigh haematoma requiring interruption of LMWH treatment. Since FUBCs proved positive again for KPC-Kp isolate fully susceptible to CZA (MIC ≤ 2 μg/mL), on day 121 colistin was replaced with CZA. However, on day 135 the patient was still febrile and PCT increased up to 25.68 ng/mL (normal value <0.5 ng/mL). Thus, after obtaining Ethical Committee approval and relative’s informed consent, MVB 2 g/2 g every 8 h (3-h infusion) was started on compassionate basis with prompt clinical improvement and clearance of bacteremia within 9 days. Interestingly, rectal swab for CRE also turned negative during MVB treatment. Meanwhile, a new CT angiography showed a complete resolution in the right subclavian vein and a significant reduction of the thrombosis in the left one. Furthermore, thanks to the healing of the thigh haematoma, the anticoagulation treatment could be restarted.

On day 145, plasma concentrations were 2.72 μg/mL for MEM and 7.07 μg/mL for vaborbactam before dose administration; 5.66 μg/mL for MEM and 23.38 μg/mL for vaborbactam after dose administration.

On day 163, MVB was discontinued and a new Doppler ultrasonography performed on day 167 revealed an almost complete revascularization of the left subclavian vein. On day 168, the patient developed candidemia: 2 days later a report was available that KPC-Kp was again isolated from FUBCs. MVB and FOS were administered to complete a 14-day treatment course and the new CVC was again removed but culture of the tip yielded no microbial growth.

On day 181, plasma concentrations were 2.27 μg/mL for MEM and 6.45 μg/mL for vaborbactam before dose administration; 9.65 μg/mL for MEM and 29.00 μg/mL for vaborbactam after dose administration. Moreover, on the same day plasma concentrations of FOS were 185.95 μg/mL and 1296.03 μg/mL before and after dose administration, respectively.

The patient underwent prompt clinical improvement with rapid bacteremia clearance (48 h). Anticoagulant treatment with LMWH was further maintained until a new Doppler ultrasonography performed on day 210 showed complete revascularization of both subclavian veins.

On day 219, the patient was discharged and transferred into a long-term care unit. No infection relapse was observed after a 3-month follow-up period.

**Figure 2:**
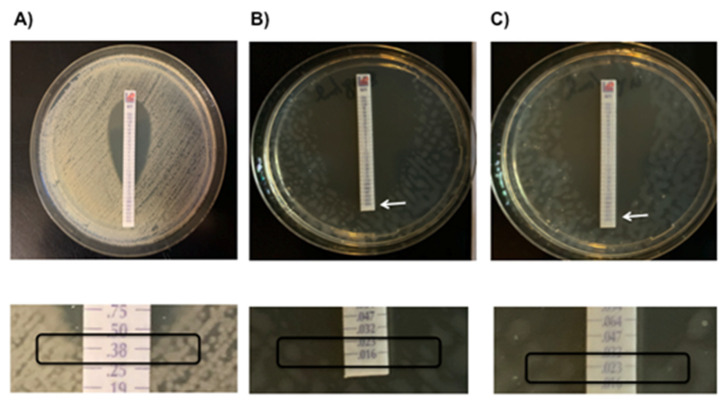
In vitro analyses of MVB (**A**) and MVB + FOS (**B**,**C**) against KPC-Kp strain isolated from the blood. Gradient strip test (E-test) showed MVB MIC of 0.38 μg/mL (Panel A). Synergy tests showed that, in the presence of 0.5×MIC (8 μg/mL) and 0.25×MIC (4 μg/mL) of FOS, MVB MICs lowered to 0.016 and 0.023 μg/mL, respectively. FOS MIC was determined with agar dilution method and was 16 μg/mL (image not shown). Arrows indicate MVB MIC in the presence of FOS. MVB: meropenem/vaborbactam; FOS: fosfomycin. In the lower part of the figure, there is a magnification of the MVB MIC value in the presence of FOS.

On day 137, KPC-Kp blood isolate was submitted to additional in vitro analyses, showing MVB, CZA and FOS MICs of 0.38 (Figure 2, Panel A), and 0.75 and 16 μg/mL, respectively. Synergy tests showed that, in the presence of 0.5×MIC (8 μg/mL) and 0.25×MIC (4 μg/mL) of FOS, MVB MICs lowered to 0.016 and 0.023 μg/mL, respectively (Figure 2, Panel B–C). MVB 0.016 μg/mL + FOS 4 μg/mL corresponded to a FICI of 0.31, thus showing full synergism.

## 3. Discussion

Herein, for the first time a case of KPC-Kp ST unresponsive to CZA and successfully treated first with MVB monotherapy and subsequently with MVB + FOS combination is presented. The case is of particular interest because of several reasons. The case is different from the condition of classical thrombophlebitis, which refers to the *ab-externo* inflammation of a thrombosed vein that always requires surgical debridement [6]; ST may be cured with prolonged targeted antimicrobial therapy and anticoagulation [1,3,4,5]. GNB ST in particular represents a subtle and often misleading condition, with rapid clinical improvement once targeted antibiotic treatment commences, despite remarkable delay of bacteremia clearance [9,10,14,15]. As a matter of fact, if FUBCs are not performed, the treating physician may fail to recognize the ST, mistakenly considering the patient cured, and then discontinue antimicrobials too soon.

In the present case, the development of a large spontaneous hematoma imposed the withdrawal of anticoagulation and this event may have had significantly impaired treatment efficacy [16]. This aspect might have contributed to the risk of CZA underexposure on the infected site and, accordingly, to its sub-optimal efficacy, reduction of activity, and therapeutic failure [17,18]. Moreover, prolonged and persistent bacteremia under CZA might have contributed to the risk of in vivo resistance development [19], confirmed by a progressive reduction of in vitro CZA activity, from full susceptibility (MIC ≤ 2 μg/mL) to a value close to the resistance breakpoint (MIC 8 μg/mL) (Figure 1).

Accordingly, with a presumptive C_min_ of 26.9 ± 15.6 and 3.5 ± 2.3 μg/mL for ceftazidime and avibactam, respectively [20], and blood isolate with an MIC of 8 μg/mL, obtaining the optimal PK/PD index (C_min_/MIC >3.8) to suppress further progression to full beta-lactams resistance [21] would have been extremely complex. For this reason, a combined colistin plus FOS treatment regimen was initiated. Interestingly, this regimen proved unsuccessful but blood isolates returned to being fully susceptible to CZA. Following MVB therapy, which was also chosen because the isolate was KPC producing, a prompt clinical improvement and bacteremia clearance were finally observed. The decision to interrupt MVB after 28 days of therapy was mainly based on the low availability of MVB supply. Furthermore, we were also confident that treatment of ST was successful as almost complete revascularization of the left subclavian vein was observed at ultrasonography and that anticoagulation could have been restarted. However, following MVB interruption, a KPC-Kp BSI relapse was observed, which was successfully treated with the innovative association of MVB plus FOS. Since culture of the removed CVC tip yielded no microbial growth, the KPC-Kp BSI relapse might have been the result of a residual ST and not just a CVC-related BSI. Thus, we decided to use MVB + FOS combination therapy, which resulted extremely synergistic in in vitro studies. Of interest, pharmacokinetic analyses also showed that MVB was in the therapeutic range both in mono and combination therapy.

Prospective controlled comparative studies of CZA and MVB in patients with severe infections caused by CRE are presently lacking. MVB may have a higher barrier to resistance compared with CZA, as vaborbactam has the ability to overcome D179Y mutation at the KPC binding site, which usually confers resistance to CZA [21]. Ackley et al. [22] recently reported a multicenter retrospective cohort of adults with CRE infections. Interestingly, although safety and efficacy outcomes were similar, a post hoc analysis showed a progressive reduction in susceptibility in six patients that received CZA and in no patient in the MVB arm. More important, development of CZA resistance was associated to recurrent infection in three patients [22].

The in vivo synergistic antimicrobial effect of MEM plus FOS in Gram-negative infections is demonstrated even on KPC-Kp strains, probably related to their activity at different stages of the peptidoglycan synthesis [23]. Although expected, no data are available in the literature on synergism between MVB and FOS. As a matter of fact, our in vitro study showed a remarkable synergistic activity of the combination of MVB plus FOS, making the present report, to the best of our knowledge, the first demonstrating synergy between these two antimicrobials.

## 4. Materials and Methods

### 4.1. In Vitro Studies

On day 137, KPC-Kp blood isolate was submitted to additional in vitro analyses including MVB, CZA, and FOS susceptibilities followed by MVB + FOS synergy tests. A gradient strip test (E-test) was used for MVB and CZA minimal inhibitory concentrations (MICs), whereas for FOS agar dilution method with supplementation of 25 mg/liter of glucose-6-phosphate was performed [24]. For synergy tests, we determined the MVB MIC by E-test on agar plates containing fixed concentrations of FOS (0.25×MIC, 0.5×MIC, with FOS MIC considered 16 μg/mL as resulted by agar dilution) and then calculated the resulting MVB MIC and FIC index (FICI). A synergistic interaction was defined as FICI ≤ 0.5. Experiments were performed in duplicate.

### 4.2. Pharmacokinetic Analyses

Blood samples for pharmacokinetic analyses were collected on day 145 (MVB monotherapy) and on day 181 (MVB plus FOS), before and after antibiotics administration. Plasma was separated by centrifugation at 3500× *g* for 5 min and stored at 80 °C until analysis. The plasma levels of MEM were determined by high performance liquid chromatography (HPLC), the extraction procedure and HPLC analysis were performed as reported by Cairoli et al. [25]. The plasma levels of vaborbactam and FOS were determined by liquid chromatography and mass spectrometry (UHPLC) Agilent 1290 Infinity II 6470 (Agilent Technologies, Waldbronn, Germany) equipped with an ESI-JET-STREAM source operating in the positive ion (ESI+) mode for FOS and in the negative ion (ESI−) mode for vaborbactam. The software used for controlling this equipment and analyzing data was MassHunter Workstation (Agilent Technologies, Waldbronn, Germany). The separation column was Zorbax Eclipse Plus C18 RRHD 1.8 μm 50 × 2.1 mm (Agilent Technologies, Santa Clara, CA, USA) at a flow rate of 0.3 mL/min for FOS and Poroshell HPH C18 2.7 μm 2.1 × 100 mm (Agilent Technologies, Santa Clara, CA, USA) at a flow rate of 0.4 mL/min for vaborbactam. Samples for determination of FOS and vaborbactam were extracted as follows: 50 μL of plasma sample were added to 250 μL of acetonitrile, vortexed at least 30 s, and then centrifuged at 13,000 rpm for 9 min. 1 μL of surnatant was injected into the column. Method validation was performed based on the U.S. Food and Drug Administration guideline [26].

## 5. Conclusions

In conclusion, management of GNB ST remains a tricky clinical challenge, especially when a MDRO is involved. In our patient, MVB appeared more effective than CZA in clearing KPC-Kp bacteremia. Of interest, definitive resolution of the endovascular infection might have been due to the synergistic interaction of MVB with FOS, which possibly allowed adequate pharmacokinetic exposures of the offending pathogen to both these antibiotics.

## Figures and Tables

**Table 1:** Antimicrobial susceptibilities (MIC, µg/mL *) of serial KPC-Kp blood isolates.

Antimicrobial Agent	Day 13	Day 38	Day 105	Day 137
Amikacin	≤8	≤8	≤8	≤8
Cefepime	>8	>8	>8	>8
Ceftazidime/Avibactam	≤2	8	8	≤2
Ceftolozane/Tazobactam	>4	>4	>4	>4
Colistin	≤2	≤2	≤2	≤2
Ertapenem	1	>1	>1	>1
Gentamicin	>4	>4	>4	>4
Imipenem	>8	>8	>8	>8
Levofloxacin	>1	>1	>1	>1
Meropenem	8	32	32	32
Aztreonam	>4	>4	>4	>4
Piperacillin/Tazobactam	>16	>16	>16	>16
Trimetoprim/Sulfametoxazole	>4/76	>4/76	>4/76	>4/76
Tigecycline	≤1	≤1	≤1	≤1
KPC **	POS	POS	POS	POS

* Minimal inhibitory concentrations (MICs) were determined by an automated microdilution technique (MicroScan WalkAway 96 Plus, Beckman Coulter, Brea, CA, USA); ** the presence of the *bla*_KPC_-gene was accomplished by RT-PCR using the GeneXpert System (Cepheid, Sunnyvale, CA, USA).

## Data Availability

Additional data are available from the corresponding author upon request.

## Abbreviations

KPC: *Klebsiella* producing carbapenemase; GNB-ST: Gram-negative bacilli septic thrombosis; FOS: fosfomycin; KPC-Kp: KPC-producing *K. pneumoniae*; CZA: ceftazidime/avibactam; MVB: meropenem/vaborbactam; ST: septic thrombosis; MDRO: multi-drug resistant organisms; MRSA: methicillin-resistant *Staphylococcus aureus*; CRE: carbapenem-resistant *Enterobacterales*; BT: body temperature; BSI: bloodstream infection; BCs: blood cultures; FUBCs: follow up blood cultures; CVC: central venous catheter; LMWH: low molecular weight heparin; UI: international units; PCT: procalcitonin; MIC: minimal inhibitory concentration; CT: computer tomography; MEM: meropenem; MU: million units; FIC: fractional inhibitory concentration; FICI: FIC index; E-test: gradient strip test; PK: pharmacokinetic; PD: pharmacodynamic; Cmin: minimum serum concentration; HPLC: high performance liquid chromatography; UHLPC: liquid chromatography and mass spectrometry; ESI+: ESI-JET-STREAM source operating in the positive ion.
